# Trampolinspringen-induzierte Makrohämaturie: Das demaskierte Nussknackersyndrom

**DOI:** 10.1007/s00120-022-01825-4

**Published:** 2022-04-14

**Authors:** Alexander Cox, A. Hofmann, C. Neissner, C. Federle, W. H. Rösch

**Affiliations:** 1 Klinik fürUrologie und Kinderurologie, Universitätsklinikum Bonn, Venusberg-Campus 1, 53127 Bonn, Deutschland; 2 Klinik für Kinderurologie, Klinik St. Hedwig in Kooperation mit der Universität Regensburg, Regensburg, Deutschland; 3 Klinik für Gastroenterologie und Interventionelle Endoskopie, Krankenhaus Barmherzige Brüder, Regensburg, Deutschland

**Keywords:** Nussknackersyndrom, Duplexsonographie, Flankenschmerz, Nierenvene, A. mesenterica superior, Nutcracker syndrome, Duplex ultrasound, Flank pain, Renal vein, Superior mesenteric artery

## Abstract

Es wird über den Fall eines 12-jährigen Jungen mit sportinduzierter, rezidivierender Makrohämaturie und linksseitigen Flankenschmerzen berichtet. Nach umfangreicher laborchemischer sowie bildgebender Diagnostik wird in Zusammenschau des charakteristischen klinischen Bildes die Diagnose Nussknackersyndrom gestellt. Unter konservativen Maßnahmen sowie Verzicht auf den auslösenden Sport kam es zu einer klinischen sowie bildmorphologisch bestätigten Maturation.

## Abstract (en)

The case of a 12-year-old boy with sports-induced recurrent macrohematuria and left-sided flank pain is reported. After extensive laboratory and imaging diagnostics, the diagnosis of nutcracker syndrome is made based on the characteristic clinical manifestation. Under a conservative approach and abstention from the triggering sport, a clinical as well as image-morphologically confirmed maturation occurred.

## Falldarstellung

### Anamnese

Ein 12-jähriger Junge wurde aufgrund einer rezidivierenden Makrohämaturie mit linksseitigen Flankenschmerzen in unserer Kinderurologie vorstellig. Extern wurde eine Urolithiasis mittels Steinsuche-CT bereits ausgeschlossen. Die Anamnese hinsichtlich Traumata, Infektionen, Diarrhöen, Gerinnungsstörungen, Manipulationen oder Medikamenteneinnahmen war unauffällig. Bereits 2 Jahre zuvor ist es zu einer ähnlichen Symptomatik gekommen. Damals sei die oben genannte Symptomatik aus linksseitigen Flankenschmerzen und Makrohämaturie durch wiederholtes „Salto-Schlagen“ im Meerwasser ausgelöst worden. Zum Zeitpunkt der Vorstellung in unserer Klinik sprang der Junge weiterhin leidenschaftlich gerne Trampolin. Im Rahmen der beiden Episoden habe er zusätzlich einen deutlichen Wachstumsschub durchgemacht.

### Klinischer Befund

Bei Vorstellung bestanden akute linksseitige Flankenschmerzen, die unter analgetischer Therapie suffizient behandelt waren. Zudem kam es unter den Schmerzepisoden wiederholt zu einer ausgeprägten Makrohämaturie mit Koagelbildung. Die körperliche Untersuchung war bei schlankem Ernährungszustand im Wesentlichen unauffällig. Fieber habe zu keinem Zeitpunkt bestanden. Ödeme oder ein arterieller Hypertonus lagen nicht vor.

### Diagnose

#### Labor

Das Aufnahmelabor war blande, es lagen keine Anämie, Infekt- oder Retentionsparametererhöhung sowie kein Anhalt für einen Diabetes mellitus vor. Der Gerinnungsstatus war unauffällig. Ein von-Willebrand-Syndrom wurde ausgeschlossen. Das C3- und C4-Kompliment sowie der Antistreptolysin-O-Titer im Serum waren normwertig. Die ANCA (antineutrophile zytoplasmatische Antikörper) Anti-PR3 (Antiproteinase‑3, c‑ANCA) sowie Anti-MPO (Myeloperoxidase, p‑ANCA) waren negativ.

#### Urinstatus

Im bei Aufnahme durchgeführten Urinstatus fanden sich eine Mikrohämaturie und Proteinurie bei minimaler Leukozyturie. Die Urinkultur blieb steril.

#### 24-h-Sammelurin

Der am 3. Aufnahmetag unter bereits sistierter Makrohämaturie durchgeführte 24-h-Sammelurin ergab eine unauffällige Proteinausscheidung (< 0,04 g/l). Der Kreatinin-Eiweiß-Quotient war im normwertigen Bereich.

#### Urosonographie

**Figure 1:**
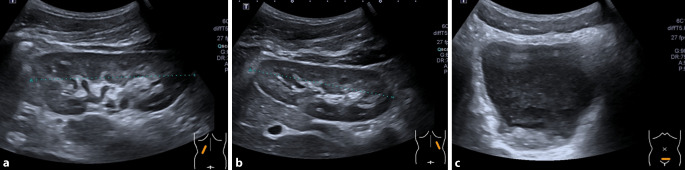

Die Nieren waren beidseits ohne Hinweis auf eine Ektasie, ein Konkrement oder eine solide Raumforderung. In der Harnblase fand sich eine Sedimentation mit Verdacht auf eine Koagelbildung (Abb. 1).

#### MRT des Abdomens am Aufnahmetag

In der MRT des Abdomens konnte eine Raumforderung als Blutungsursache ausgeschlossen werden. Es konnten jedoch bildmorphologische Zeichen eines „anterioren Nussknackersyndroms“ dargestellt werden.

#### MR-Angiographie

**Figure 2:**
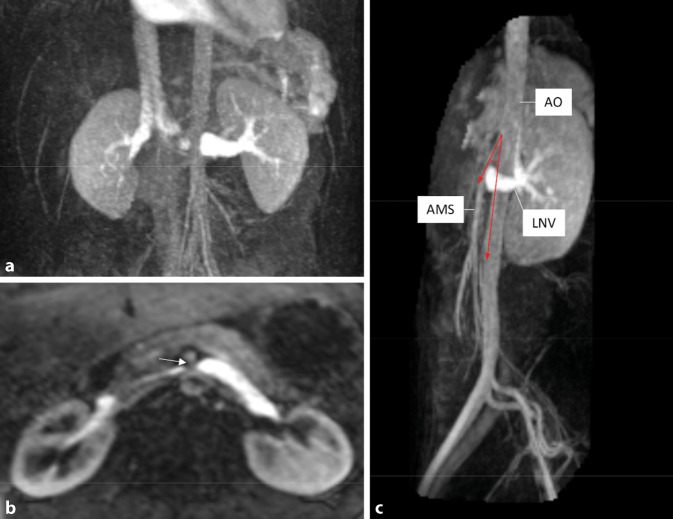

Im weiteren Verlauf erfolgte bei persistierender intermittierender Makrohämaturie ergänzend eine MR-Angiographie zum Ausschluss einer Gefäßmalformation. Eine arteriovenöse Fistel wurde ausgeschlossen, jedoch bestätigten sich die bildmorphologischen Zeichen eines Nussknackerphänomens. Hierbei wird die linke Nierenvene zwischen der anterior verlaufenden A. mesenterica superior und der dorsal gelegenen Aorta abdominals eingeengt, wodurch der venöse Abfluss der linken Niere kompromittiert wird (Abb. 2a, b). Bei unserem Patienten betrug der Abgangswinkel der A. mesenterica superior aus der Aorta ca. 20° (normal > 45°; Abb. 2c).

#### Duplexsonographie

**Figure 3:**
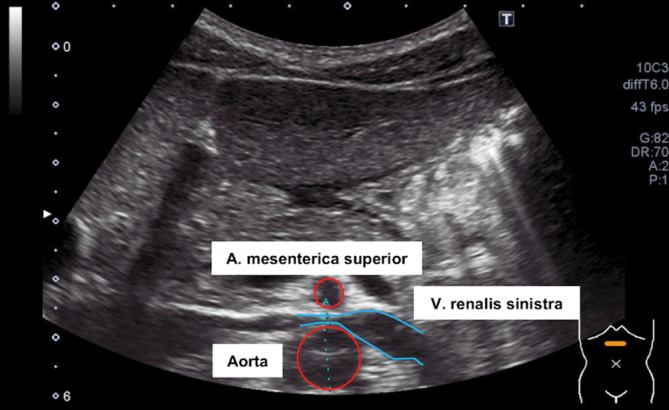

Da das beschriebene Nussknackersyndrom nicht selten als Zufallsbefund in einer Schnittbildgebung beschrieben wird, erfolgte ergänzend eine Duplexsonographie. Sowohl die knapp 6fach höhere Flussgeschwindigkeit im komprimierten Anteil der Nierenvene (100 vs. 17 cm/s; Norm: < 4:1; [1]) als auch das Verhältnis der Diameter von 4,1:1 (5,7 vs. 1,4 mm; Norm < 4,2:1 bzw. < 3,7:1; [2]) sprachen für ein relevantes Nussknackersyndrom (Abb. 3).

### Therapie und Verlauf

Die häufigsten Ursachen der Symptomkombination aus Makrohämaturie und Flankenschmerz wie Trauma, Harnwegsinfektion, Urolithiasis und Tumoren wurden zunächst ausgeschlossen. Eine Gerinnungsstörung wie z. B. das von-Willebrand-Syndrom lag ebenfalls nicht vor. Nach konsiliarischer kindernephrologischer Vorstellung konnte eine für die Symptome ursächliche Glomerulonephritis (im Speziellen IgA-Nephritis, postinfektiöse Glomerulonephritis und Glomerulonephritiden im Rahmen von Vaskulitiden wie Morbus Wegener, mikroskopische Polyangitis und Purpura Schönlein-Henoch) ausgeschlossen werden. Auf eine Urethrozystoskopie zum Ausschluss der wichtigen Differentialdiagnose einer Makrohämaturie bei präpubertären Jungen, der Urethritis posterior, wurde verzichtet, da das Ausmaß der Makrohämaturie mit Koagelbildung sowie die akuten kolikartigen Flankenschmerzen als nicht pathognomonisch für diese Erkrankung zu bewerten waren. Nach Ausschluss dieser relevanten Differentialdiagnosen sprach die Kombination aus dem bildmorphologischen Nussknackerphänomen und den charakteristischen Symptomen für das Vorliegen eines Nussknackersyndroms. Bei deutlicher Besserung der Beschwerden unter körperlicher Schonung sowie adäquater Analgesie, jungem Alter sowie stabiler Nierenfunktion wurde ein konservatives Management vereinbart. Neben einer Gewichtszunahme wurde zu einem Verzicht auf Trampolinspringen geraten. Hierunter ist der junge Patient nun seit 12 Monaten beschwerdefrei. Zur Objektivierung der Befunde erfolgte ein Jahr nach Vorstellung eine duplexsonographische Kontrolle, in der eine Maturationstendenz bestätigt werden konnte. Das Verhältnis der Diameter des komprimierten zum dilatierten Anteil der Nierenvene lag nun bei 1,8:1 (4,5 vs. 2,5 mm). Lediglich das Verhältnis der Flussgeschwindigkeiten war mit 4,2:1 (71 vs. 17 cm/s) noch grenzwertig erhöht, insgesamt jedoch deutlich gebessert.

## Diskussion

Aufgrund der variablen klinischen Präsentation sowie fehlendem, einheitlichem Konsens zu diagnostischen Kriterien lässt sich derzeit keine genaue Aussage zur Prävalenz des Nussknackersyndroms treffen. Die höchste Inzidenz findet sich in der zweiten Lebensdekade sowie im mittleren Erwachsenenalter [3]. Das rasche Wachstum sowie die Entwicklung der Wirbelkörper in der Pubertät scheinen einen spitzen aortomesenterialen Winkel zu begünstigen. Dieses klassische, anteriore Nussknackersyndrom wird vom sehr seltenen posterioren Syndrom, bei dem es zu einer Kompression der Nierenvene zwischen der Aorta und dem Wirbelkörper kommt, unterschieden. Die aus beiden Formen resultierenden Symptome sind jedoch identisch. Neben den klassischen Symptomen Mikro‑/Makrohämaturie, Flanken- und/oder Beckenschmerzen sowie Proteinurie kann die venöse Stauung auch zur Varicocele testis bzw. im fortgeschrittenen Stadium insbesondere bei Frauen zum pelvinen Stauungssyndrom führen. Im hier geschilderten Fallbeispiel lag allerdings keine Varikozele vor. Die Hämaturie wird nach aktuellem Stand auf eine reflux- und druckbedingte Ausbildung von peripelvinen und -ureteralen Varizen, die einreißen können, zurückgeführt [4]. Diese Hypothese steht im Einklang mit den Symptomen des betroffenen Jungen: Es ist anzunehmen, dass die ausgeprägte Makrohämaturie durch eine Akzeleration im Rahmen der Salti bzw. des Trampolinspringens ausgelöst wurde. Eine Lage- und Bewegungsabhängigkeit der Hämaturie ließ sich auch im stationären Aufenthalt des Patienten in geringem Ausmaß klinisch reproduzieren. Die Ausprägung der im Sediment nachgewiesenen Mikrohämaturie war gegen Nachmittag stets deutlich ausgeprägter als am Morgen.

Das Nussknackersyndrom stellt häufig eine Ausschlussdiagnose dar, seine Diagnostik ist vielseitig und nicht selten herausfordernd. Bei ausreichend vorhandener Expertise sollte die Duplexsonographie in der Primärdiagnostik als Mittel der Wahl eingesetzt werden. Zwei Messungen sind hierbei obligat: das Verhältnis des systolischen Spitzenflusses im komprimierten sowie dilatierten Abschnitt der Nierenvene sowie des jeweiligen Diameters. In der Literatur variieren die Grenzwerte zwischen 4,0:1–5,0:1 [1, 5] bzw. 3,7:1–4,2:1 [2]. Bei unzureichendem Ergebnis kann eine Schnittbildgebung mittels MRT oder CT erfolgen, in deren axialen und koronaren Aufnahmen die Kompression der Vene bzw. der Abgangswinkel der A. mesenterica superior sowie sekundären Genesen demonstriert werden können. Als Grenzwert für das Verhältnis der Diameter wurde 4,9:1 festgelegt [6]. In der Literatur wurden auch Referenzwerte zum aortomesenterialen Abgangswinkel der A. mesenterica superior definiert, die ein symptomatisches Nussknackerphänomen, das „Nussknackersyndrom“, wahrscheinlich machen: ein Winkel von > 45° stellt in der Regel ein Normalbefund dar, wohingegen ein Winkel von < 35° signifikant für ein Nussknackersyndrom erscheint [4]. Aufgrund der wachsenden Verfügbarkeit moderner MRT ist der ehemalige, invasive Goldstandard der Venographie zur Bestimmung des renokavalen Druckgradienten nur noch selten indiziert. Ein Druckgradient von > 3 (Norm: 0–1) mmHg soll die Diagnose sichern [7]. Allerdings wurden auch bei Patienten mit relevantem Nussknackersyndrom Druckgradienten von < 3 mmHg gemessen, was durch die Ausbildung von venösen Kollateralen, die die venöse Hypertonie kompensieren, erklärt wird. Entsprechend sollte das Nussknackersyndrom stets unter Berücksichtigung charakteristischer Symptome bewertet werden.

Ebenso weitreichend wie die Diagnostik sind die therapeutischen Optionen. Bei ausbleibendem Ansprechen auf eine konservative Therapie oder schwerer Symptomatik wie eine Nierenvenenthrombose, therapierefraktäre Schmerzen oder eine rezidivierende, Hb-relevante Makrohämaturie kann eine offen-chirurgische Transposition der linken Nierenvene oder eine in den letzten Jahren frequentiert durchgeführte endovaskuläre Stenteinlage erfolgen. Die genannten Verfahren erzielen insgesamt gute Ergebnisse, weisen jedoch nicht unerhebliche Risiken auf. Neben den typischen Risiken offen-chirurgischer Eingriffe sind Thrombosen und Dislokationen des Stents zu nennen. Darüber hinaus ist zumeist eine mindestens 3‑monatige Antikoagulation nach Stenteinlage indiziert. Das pädiatrische Patientenklientel sowie Patienten mit geringer Symptomatik sollten demzufolge primär eine konservative Therapie erhalten. Der Beobachtungszeitraum bei Kindern sollte hierbei mindestens 24 Monate betragen, da eine Spontanmaturationsrate von > 75 % besteht. Ursächlich für die Maturation erscheint die Zunahme des retroperitonealen und mesenterialen Fettgewebes im Rahmen des Wachstums, wodurch der Abgangswinkel der A. mesenterica superior vergrößert und die dorsale Ptose der Niere reduziert wird [8]. Diese Hypothese wird gestützt durch eine negative Korrelation des Body Mass Index (BMI) mit der Spitzenflussgeschwindigkeit in der komprimierten Vene sowie einer Symptomreduktion mit steigendem BMI [9].

## Fazit für die Praxis

- Das Nussknackersyndrom ist eine seltene Differentialdiagnose einer Makrohämaturie, das häufig protrahiert diagnostiziert wird.
- Die diagnostischen Möglichkeiten sind vielfältig und variabel. Bei unklarem Befund einer Duplexsonographie kann die MR-Angiographie ergänzend eingesetzt werden.
- Die Bewertung der charakteristischen Symptome in Kombination mit der Bildgebung ist für die Diagnosestellung obligat.
- Bei hoher Spontanmaturationsrate sollte insbesondere bei Kindern eine konservative Therapie angestrebt werden.
